# The thiolation of uridine 34 in tRNA, which controls protein translation, depends on a [4Fe-4S] cluster in the archaeum *Methanococcus maripaludis*

**DOI:** 10.1038/s41598-023-32423-9

**Published:** 2023-04-01

**Authors:** Ornella Bimai, Pierre Legrand, Jean-Luc Ravanat, Nadia Touati, Jingjing Zhou, Nisha He, Marine Lénon, Frédéric Barras, Marc Fontecave, Béatrice Golinelli-Pimpaneau

**Affiliations:** 1 Laboratoire de Chimie des Processus Biologiques, Collège de France, CNRS UMR 8229, Sorbonne Université, 11 Place Marcelin Berthelot, 75231 Paris Cedex 05, France; 2 Synchrotron SOLEIL, L’Orme des Merisiers, Saint Aubin BP48, 91198 Gif-sur-Yvette, France; 3 University of Grenoble Alpes, CEA, CNRS, IRIG, SyMMES, UMR 5819, 38000 Grenoble, France; 4 IR CNRS Renard, Chimie-ParisTech, 11 rue Pierre et Marie Curie, 75005 Paris, France; 5 Stress Adaptation and Metabolism in Enterobacteria Unit, Institut Pasteur, Université Paris Cité, UMR CNRS 6047, Paris, France

**Keywords:** X-ray crystallography, Metalloproteins, Biocatalysis, Enzyme mechanisms, RNA

## Abstract

Thiolation of uridine 34 in the anticodon loop of several tRNAs is conserved in the three domains of life and guarantees fidelity of protein translation. U34-tRNA thiolation is catalyzed by a complex of two proteins in the eukaryotic cytosol (named Ctu1/Ctu2 in humans), but by a single NcsA enzyme in archaea. We report here spectroscopic and biochemical experiments showing that NcsA from *Methanococcus maripaludis* (MmNcsA) is a dimer that binds a [4Fe-4S] cluster, which is required for catalysis. Moreover, the crystal structure of MmNcsA at 2.8 Å resolution shows that the [4Fe-4S] cluster is coordinated by three conserved cysteines only, in each monomer. Extra electron density on the fourth nonprotein-bonded iron most likely locates the binding site for a hydrogenosulfide ligand, in agreement with the [4Fe-4S] cluster being used to bind and activate the sulfur atom of the sulfur donor. Comparison of the crystal structure of MmNcsA with the AlphaFold model of the human Ctu1/Ctu2 complex shows a very close superposition of the catalytic site residues, including the cysteines that coordinate the [4Fe-4S] cluster in MmNcsA. We thus propose that the same mechanism for U34-tRNA thiolation, mediated by a [4Fe-4S]-dependent enzyme, operates in archaea and eukaryotes.

## Introduction

Precise decoding of the genetic code is a fundamental process in all living organisms, with tRNAs playing a critical role in translating codon triplets on mRNAs to corresponding amino acids within the ribosome. To achieve accurate translation, tRNA molecules feature post-transcriptional chemical modifications, which have been conserved through evolution. These modifications stabilize the tRNA tertiary structure, introduce recognition determinants and anti-determinants towards RNA-interacting macromolecules and fine-tune the decoding process both in terms of efficiency and fidelity^1,2^. Interestingly, recent studies have revealed unsuspected roles of these modifications in the regulation of translation and protein homeostasis during cellular stress^3–8^.

**Figure 1:**
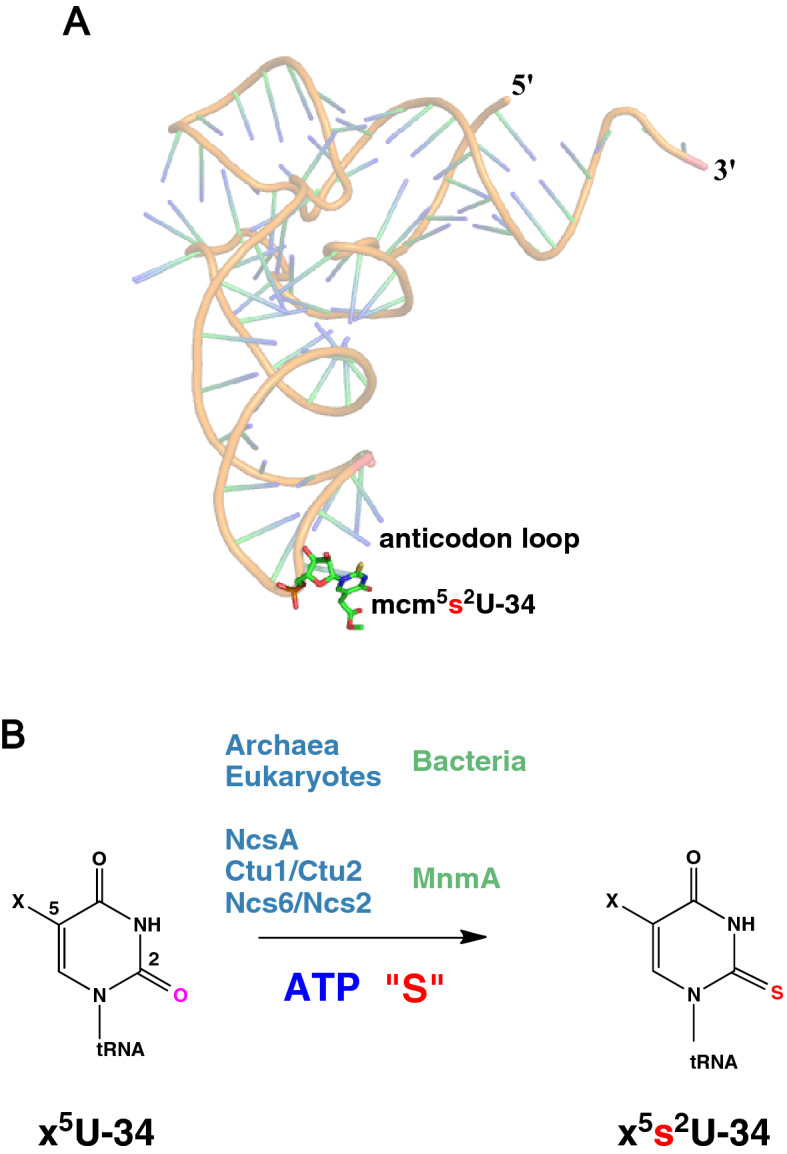
U34-tRNA thiolation. (**A**) Location of mcm^5^s^2^U34 (in stick representation) in human tRNA^Lys3^ (PDB code 1FIR). (**B**) Reaction catalyzed by U34-tRNA thiolases. The modification at the C5 position (noted x) varies upon the organism (see text).

Modifications at position 34 (wobble base, Fig. 1A) of tRNAs are crucial for translation^9,10^. Indeed, position 34 is the first base in the tRNA anticodon, which is bound less tightly to the ribosome than the two other positions of the anticodon, enabling it to participate to non-canonical base pairs. Since this loose binding enhances the probability of amino acid mis-incorporation into the growing peptide chain, complex chemical base modifications at the wobble base were selected during evolution for accurate tRNA-codon recognition^1,11,12^. For instance, U34 of tRNA^Gln^_UUG_, tRNA^Lys^_UUU_ and tRNA^Glu^_UUC_ is universally thiolated at C2 position and hypermodified by different chemical groups at C5 position depending on the organism: methylaminomethyl (mnm), carboxymethylaminomethyl (cmnm), aminomethyl (nm) or isopentenylaminomethyl (inm) in bacteria^13^, mnm and carbamoylmethyluridine (ncm) in archaea^14,15^, methoxycarbonylmethyl (mcm) in the eukaryotic cytosol, cmnm in yeast mitochondria, or taurinomethyl (τm) in mammalian mitochondria^16^. Whereas unmodified U can base pair with all of four A, U, G, C nucleosides, modification of U34 provides base pairing specificity: modification at C5 contributes to the preordering of the anticodon loop into a U-turn structure^17^ and restricts the reading to codons ending with A or G^18^, whereas the thio group at C2 stabilizes the 3′-endo conformation of the ribose^19^ and enables base pairing with G via a new wobble pattern^20,21^.

The structural basis for the specific pairing of mnm^5^s^2^U34 with the AAA and AAG codons was unveiled by comparing several crystal structures of the *Thermus thermophilus* 70S ribosome in complex with native tRNA^Lys^_UUU_ (containing mnm^5^s^2^U34) and mRNA, in which either the cognate (AAA or AAG) or near-cognate (UAA or AUA) codon was present in the A-site^21^. The unusual base pair between mnm^5^s^2^U34 and guanosine was shown to result from the stabilization of the U-turn structure of the anticodon loop through a hydrogen bond between the mnm amino group and the 2′OH of U33, and from a stacking interaction between the thio group at U34 and the N1 atom of U35.

Biochemical studies have shown that thiolation of U34 acts as a determinant for tRNA aminoacylation and favors the initial steps of decoding on the bacterial or eukaryotic ribosome. Indeed, the binding of native *E. coli* mnm^5^s^2^U34-containing tRNA^Gln^_UUG_ to glutaminyl-tRNA synthetase GlnRS is enhanced tenfold compared to the transcript^22^. The comparison of the crystal structures of GlnRS bound to native tRNA^Gln^, or to the unmodified transcript, showed that a specific binding pocket is formed for the 2-thio group but not for the 2-oxygen, which provides a structural basis for the improved binding of native tRNA possessing s^2^U34^23^.

In *Saccharomyces cerevisiae*, the simultaneous removal of mcm^5^U and s^2^U results in synergistically increased + 1 frameshift rates that are suppressible by extra copies of tRNA^Lys^_UUU_^24^. Comparing the decoding properties of tRNAs with and without modifications at the wobble position in yeast tRNAs has shown that the s^2^ or mcm^5^ modification at U34 increases binding of the cognate tRNA to the A site of the ribosome, GTPase activation, tRNA-mRNA translocation and peptide bond formation^9,25,26^. Moreover, mcm^5^s^2^U34 modifications of tRNA^Lys^_UUU_, tRNA^Gln^_UUG_ and tRNA^Glu^_UUC_ have been shown to favor the expression of a subset of mRNAs enriched for codons that are read by these tRNAs, mediating the cellular stress response^25–27^. In humans, modifications at U34-tRNA also regulate specific mRNA translation, promoting resistance of melanoma cells to therapy^28^.

Thiolation of U34 is catalyzed by an MnmA-type enzyme in bacteria^29^ and mitochondria^30^, a complex of two proteins in the cytosol of eukaryotes, called Ncs6/Ncs2 in yeast and Ctu1/Ctu2 in higher eukaryotes^31^, but by a single protein called NcsA in archaea^32,33^ (Fig. 1B). The two proteins forming the eukaryotic complex display significant sequence homology (23% sequence identity between Ctu1 and Ctu2 and 12% sequence identity between Ncs6 and Ncs2; Fig. S1), suggesting that they evolved from a common ancestor. MnmA-type enzymes and Ncs6/Ctu1/NcsA proteins belong to two different subclasses of the PP-loop ATP pyrophosphatase family^34^. Whereas several biochemical and structural studies of bacterial MnmAs have been reported^29,35–37^, information is lacking for the second subfamily, especially at the molecular level.

Ctu1/Ncs6/NcsA proteins display close sequence similarity to members of the TtcA-TtuA family^34^ and contain the three conserved cysteines that are ligands of a [4Fe-4S] cluster in this subfamily (Fig. S1). Therefore, we hypothesized that they all bind a catalytically essential [4Fe-4S] cluster and share the same catalytic mechanism^38–42^. Like the TtuA subfamily, Ncs6/NcsA/Ctu1 proteins possess zinc-finger domains at the N and C-termini^43^ (Fig. S1).

Whereas human Ctu1 and yeast Ncs6 bearing a Maltose-Binding-Protein tag could be produced in small amounts^44,45^, the recombinant Ctu2 protein could not be obtained in soluble form. Yet, in vitro 2-thiouridine formation could be partially reconstituted using the immunoprecipitated Ctu1–Ctu2 complex, together with L-cysteine as a sulfur donor and cysteine desulfurase Nfs1p, but the activity was very low, indicating that important components were missing^31,46^. Given that all our attempts to produce a recombinant eukaryotic Ctu1/Ctu2 complex have also failed, in order to gain insights into the properties and catalytic mechanism of the eukaryotic enzymes, we relied on the study of their archaeal NcsA orthologues, which are active as stand-alone proteins. There are only two reported studies concerning archaeal NcsA proteins. First, NcsA from *Haloferax volcanii* has previously been shown to catalyze U34-tRNA thiolation in vivo^32^. Second, NcsA from *Methanococcus maripaludis* (MmNcsA) has been expressed in *Escherichia coli* and the recombinant protein purified under anoxic conditions^33^. It has been shown that MmNcsA contains a [3Fe-4S] cluster but whether it is needed for activity or not has not been specified^45^.

We report here the in vitro biochemical, spectroscopic and structural characterization of MmNcsA, which shows that the enzyme can bind a [4Fe-4S] cluster that is essential for its U34-tRNA thiolation activity. Moreover, because obtaining the crystal structure of the human Ctu1/Ctu2 complex is a long-term goal, we take advantage of the recently released structure prediction algorithm AlphaFold^47^, to calculate and analyze the three-dimensional (3D) model of the human Ctu1/Ctu2 complex and compare it to the MmNcsA crystal structure. This comparison leads us to extend our conclusions about the mechanism of archaeal NcsA enzymes to the eukaryotic Ctu1/Ctu2 or Ncs6/Ncs2 complexes that catalyze the same biosynthetic reaction.

## Results

### MmNcsA can bind a [4Fe-4S] cluster

MmNcsA was purified under aerobic conditions (Fig. S2A,B). The UV–visible spectrum of the as-purified protein displayed several weak absorption bands, which are reminiscent of an iron-sulfur cluster (Fig. S2C), suggesting that MmNcsA is an iron-sulfur cluster-containing protein. However, only ~ 0.5 Fe per monomer was present in the protein, likely due to the fact that, as often observed, the cluster is degraded during purification. The “as-purified” protein was treated with dithionite and EDTA to remove the residual cluster and obtain the apo-MmNcsA protein, which was isolated using gel filtration chromatography (Fig. S2D). Calibration of the column with protein standards (insert, Fig. S2D) indicated that it is a globular dimer of 73.4 kDa. Reconstitution of the [Fe-S] cluster was then carried out in vitro under strict anaerobic conditions by treating apo-MmNcsA with a 5-molar excess of ferrous iron and a biochemical source of sulfide, consisting of l-cysteine and a cysteine desulfurase CsdA, in the presence of dithiothreitol. After purification on a Superdex 200 gel filtration column under anaerobic conditions, a homogenous brownish solution containing the holo-MmNcsA protein was obtained (Fig. S2E,F).

**Figure 2:**
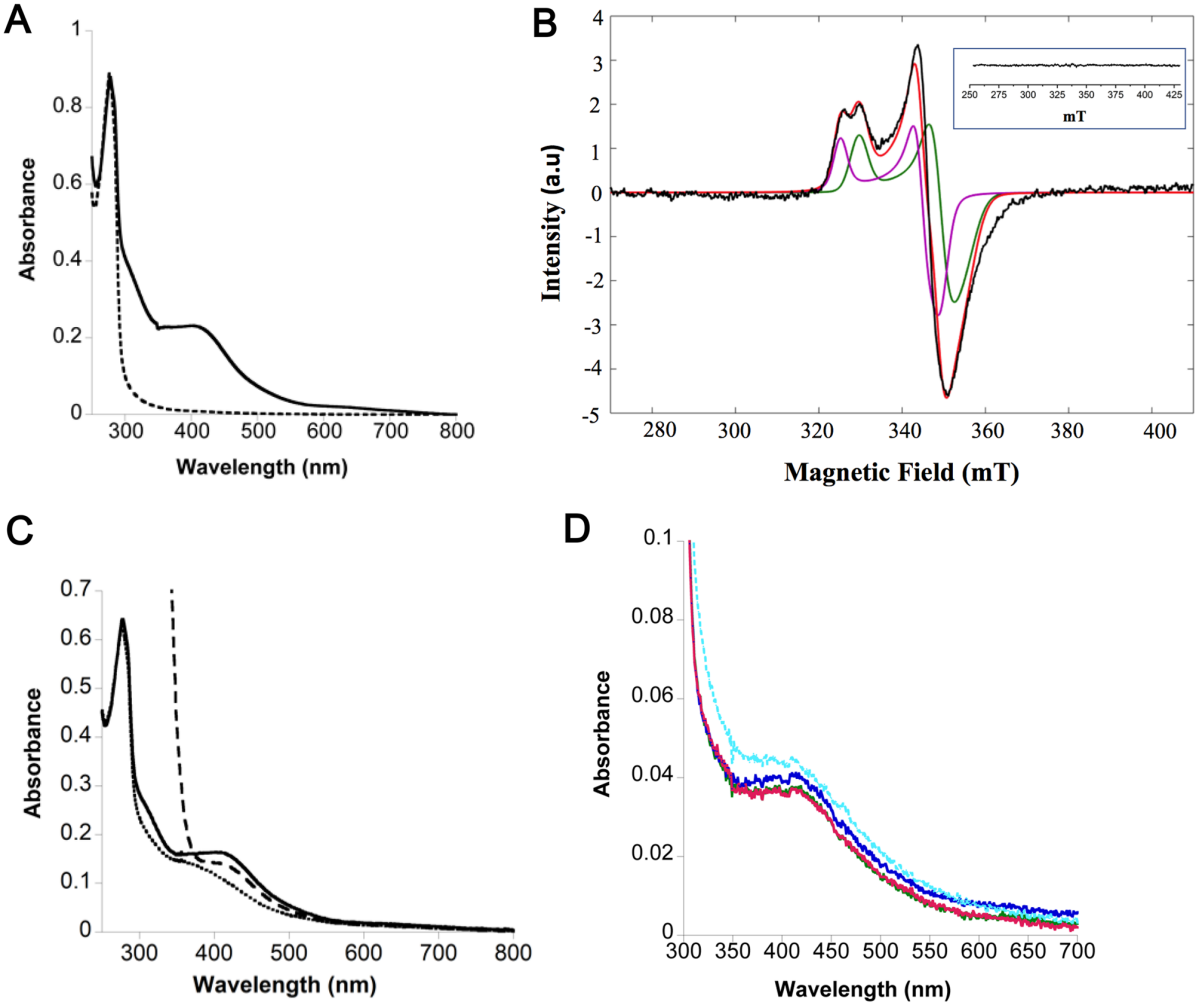
Spectroscopic characterization of MmNcsA. (**A**) UV–visible spectra of apo-MmNcsA (dashed line) and holo-MmNcsA (full line). The spectra were recorded with 40 µM protein in 25 mM HEPES pH 7.5, 200 mM NaCl. (**B**) X-band EPR spectrum of reduced holo-MmNcsA at 20 K. The buffer signal was removed from the experimental spectrum before simulation. Two clusters were simulated separately. For cluster 1 (magenta line), the 3 distinct principal values of the g-tensor are g_x_ = 1.890, g_y_ = 1.918, gz = 2.033 and the Gaussian distribution deviations σ(g_x_) = 0.03, σ (g_y_) = 0.001, σ (g_z_) = 0.002; for cluster 2 (green line), g_x_ = 1.919 g_y_ = 1.944, g_z_ = 2.061 and σ(g_x_) = 0.01, σ (g_y_) = 0,001 and σ (g_z_) = 0.001. The experimental (black line) and simulated spectra (magenta and green lines for the two cluster species with red line for their sum) are superimposed. Inset: EPR spectrum of holo-MmNcsA before reduction**.** (**C**) UV–visible spectra of holo-MmNcsA (19 µM), before (full line) and after reduction with a 25-fold excess of dithionite (dashed line) or NaBH_4_ (dotted line). (**D**) UV–visible absorption spectrum of holo-NcsA before and after reaction showing that the absorption band at 400 nm characteristic of the [4Fe-4S] cluster is not modified during the assay: 5 µM holo-NcsA just after adding 5 µM tRNA^Lys^_UUU_, 500 µM ATP, 250 µM Na_2_S and 2.5 mM MgCl_2_ (blue) and after 30 min incubation at 37 °C (cyan). A control sample containing the same reactants except Na_2_S was monitored at the beginning of the reaction (green) and after 30 min incubation (red).

Iron and labile sulfur were quantified using the Beinert and Fish methods^48,49^. The content of 3.1 ± 0.2 Fe and 4.8 ± 0.2 S per monomer agrees with the presence of one [4Fe-4S] cluster with 77.5% occupancy. Interestingly, this analysis also indicates that the protein seems to bind an additional S atom. Accordingly, purified holo-MmNcsA displayed an UV–visible spectrum with an absorption band at around 410 nm, characteristic of the presence of a [4Fe-4S]^2+^ cluster (Fig. 2A)^50^. Electron paramagnetic spectroscopy (EPR) enables to identify and quantify paramagnetic species with unpaired electron such as organics radicals or transition metals. The EPR spectrum corresponds to the first derivative of the absorption spectrum of the sample placed in a magnetic field^51^. The various types of [Fe-S] clusters display characteristic EPR spectra depending on their spin state^50,52,53^. Holo-MmNcsA was silent in continuous wave EPR spectroscopy (Inset of Fig. 2B), in agreement with the presence of an oxidized S = 0 [4Fe-4S]^2+^ cluster and the absence of any S = 1/2 [4Fe-4S]^+^ or [3Fe-4S]^+^ cluster. For further characterization by EPR spectroscopy, various attempts of cluster reduction were performed under anaerobic conditions. The cluster was resistant to reduction by treatment with 10 mM dithionite but could be partially reduced with 10 mM sodium borohydride, as shown by the decay of the absorption band at 410 nm (Fig. 2C). Indeed, the EPR spectrum of the reduced protein clearly indicated the presence of reduced S = 1/2 [4Fe-4S]^+^ species (Fig. 2B). The EPR signal at 20 K could be simulated as the sum of two distinct S = 1/2 clusters with rhombic g-tensors (g_x_ = 1.890, g_y_ = 1.918, g_z_ = 2.033) and (g_x_ = 1.919, g_y_ = 1.944, g_z_ = 2.061) (magenta and green lines, Fig. 2B), in a ratio of about 1:1, indicating the presence of two [4Fe-4S] forms with slightly different local environments.

Altogether, the Fe and S quantification combined with the spectroscopic data show that, under anaerobic conditions, MmNcsA binds a [4Fe-4S]^2+^ cluster that is quite resistant to reduction.

### The [4Fe-4S] cluster is required for the tRNA thiolation activity of MmNcsA

**Figure 3:**
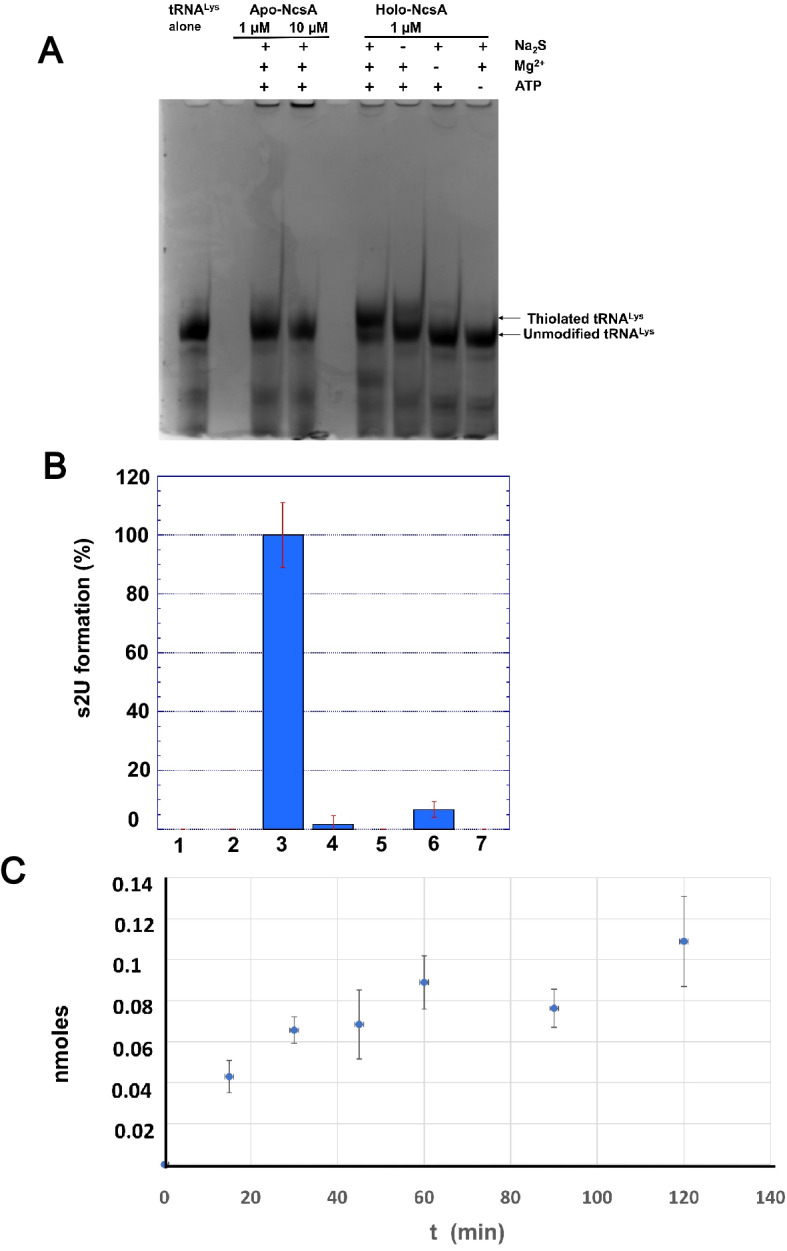
In vitro tRNA thiolation activity assays. (**A**) In vitro tRNA thiolation activity tests under anaerobic conditions monitored on an APM-retardation gel. 15 µM in vitro transcribed tRNA^Lys^_UUU_ was incubated for 1 h at 37 °C in 25 mM HEPES pH 7.5, 200 mM NaCl, with apo-MmNcsA or holo-MmNcsA, in the presence or absence of 1 mM Na_2_S, 5 mM MgCl_2_, 1 mM ATP. (**B**) Conditions for s^2^U formation monitored by HPLC-coupled MS/MS after tRNA digestion. The data shown are mean values based on 3 different experiments. (**1**) tRNA^Lys^_UUU_ alone, 10 µM apo-MmNcsA (**2**), or 1 µM holo-MmNcsA (**3**) incubated for 1 h with 20 µM tRNA^Lys^_UUU_, 2.5 mM MgCl_2_, 0.25 mM Na_2_S and 0.25 mM ATP. Control reactions: no Na_2_S (**4**), no MgCl_2_ (**5**), no ATP (**6**), U34A-tRNALys^UUU^ (**7**). 100% activity corresponds to 0.1 nmol s^2^U formed in 60 min. (**C**) Quantification of s^2^U formation catalyzed by 1 µM holo-MmNcsA incubated with 20 µM tRNA^Lys^_UUU_ in the presence of 0.25 mM ATP, 0.25 mM sodium sulfide and 2.5 mM MgCl_2_ monitored by HPLC-coupled MS/MS after tRNA digestion.

The thiolation activity of MmNcsA was tested using in vitro transcribed tRNA^Lys^_UUU_ from *M. maripaludis* (Mm-tRNA^Lys^_UUU_) as a substrate in the presence of inorganic sulfide, ATP and Mg^2+^. The activity was first monitored on a denaturing urea PAGE gel containing ((N-acryloylamino)phenyl)mercuric chloride (APM), which specifically retards migration of thio-containing molecules and thus separates thiolated tRNAs from unmodified tRNAs (Fig. 3A)^54^. Holo-MmNcsA, inorganic sulfide, ATP and Mg^2+^ were required for full production of thiolated tRNAs. Yet, we observed residual s^2^U formation in the control sample lacking the sulfur donor Na_2_S (Fig. 3A, lane 5). Importantly, apo-MmNcsA (1 or 10 µM) did not catalyze the thiolation of tRNA (15 µM), thus showing the key role of the [4Fe-4S] cluster of MmNcsA for catalysis (Fig. 3A). However, we excluded the [4Fe-4S] cluster as the source of sulfur atoms for the thiolation reaction since monitoring the fate of the cluster during catalysis by light-absorption spectroscopy did not show any degradation of the [4Fe-4S] cluster on the catalysis time scale (Fig. 2D).

To quantify the activity of MmNcsA, the tRNA products from the activity assays were digested by nuclease P1. The resulting C2-thiolated uridines were first identified by their elution position after HPLC–MS, then quantified by MS/MS (Fig. 3B,C) by monitoring the spectrum of the fragment corresponding to the loss of sugar (-132), and comparing it to the spectrum of a synthetic s^2^U standard (Fig. S3)^55^. A small amount of s^2^U (~ 1.6% or ~ 7%) could be detected in the control samples lacking the sulfur donor Na_2_S or ATP, respectively (Fig. 3B). Whereas the controls containing no enzyme, no Mg^2+^ or apo-MmNcsA showed no formation of modified nucleosides (Fig. 3B), s^2^U could be detected and was formed in a time-dependent manner when holo-MmNcsA was incubated in the presence of all reactants (Fig. 3C). Furthermore, when the tRNA^Lys^_UUU_ transcript bearing the U34A mutation was used as substrate, no s^2^U could be detected (Fig. 3B, lane 7), indicating that MmNcsA targets U at position 34 in the anticodon loop.

### Crystallographic characterization of the [4Fe-4S] cluster

**Figure 4:**
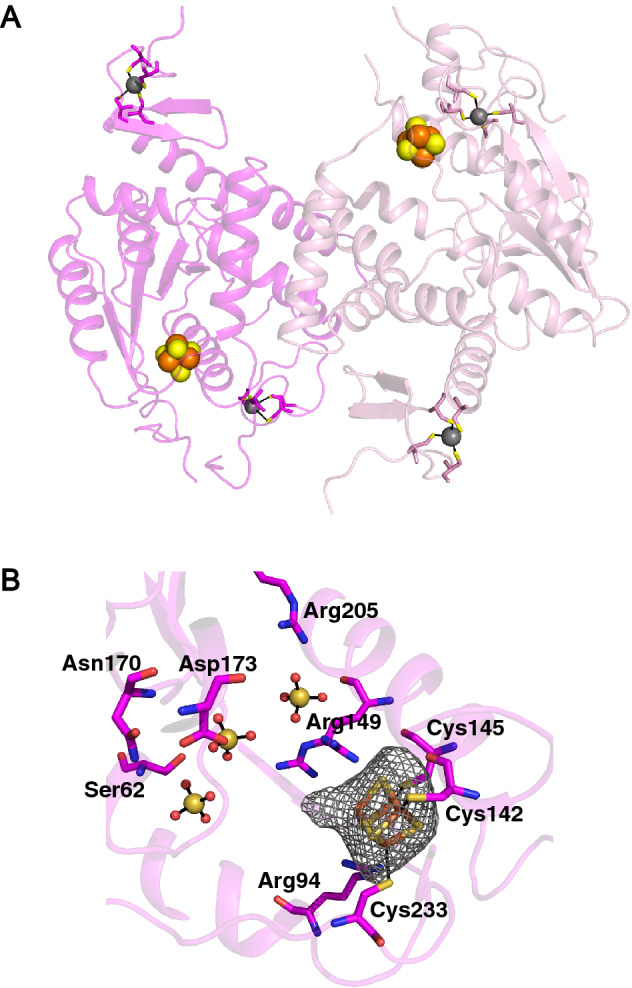
Crystal structure of holo-MmNcsA. (**A**) Overall view of the dimer. The cluster of MmNcsA is shown as orange (iron) and yellow spheres (sulfur). The zinc atoms are shown as grey spheres. MmNcsA-A is shown in pink; MmNcsA-B in magenta. (**B**) Zoom showing the [4Fe-4S] cluster in molecule A bound by 3 cysteines only, at the active site of holo-MmNcsA with a superposed Fo-Fc difference map omitting the [Fe-S] cluster contoured at 3 σ (in grey). Sulfate molecules near the cluster are depicted as spheres.

We have obtained crystals of holo-MmNcsA diffracting at 2.8 Å resolution and solved the structure by molecular replacement, using the m^5^U54 tRNA thiolase TtuA from *P. horikoshii* (PhTtuA)^39^ that shares 30% sequence identity with MmNcsA (see sequence alignment in Fig. S1) as a model (Table 1). The crystals belong to space group *P2*_*1*_*2*_*1*_*2*_*1*_ and contain two molecules in the asymmetric unit that form a dimer (Fig. 4A). The MmNcsA structure reveals three different metal sites on each polypeptide chain: one [4Fe-4S] cluster, and two mononuclear sites at the N- and C-termini, chelated by four cysteine/histidine ligands, that are presumably Zn ions, by homology with PhTtuA^56^. The [4Fe-4S] cluster is chelated by three conserved cysteines only: Cys142, Cys145, and Cys233 (Fig. 4B). Remarkably, while three Fe atoms are attached to the protein via these 3 cysteines, the fourth iron completes its coordination with a non-protein, exogenous ligand, as revealed by an electron density adjacent to that Fe atom. Based on the diffraction data at only one wavelength, it is not possible to distinguish between a water molecule, a hydrogenosulfide ion, a chloride ion or the oxygen atom of a sulfate molecule, which all could be refined with B-factors in the same range as those for the protein. Note that several sulfate ions originating from the crystallization solution were observed within the active site (Fig. 4B). A negatively charged ligand on the fourth iron atom is very likely, given its charge complementary with the [4Fe-4S]^2+^ cluster and its proximity with the guanidinium group of Arg149, located less than 3 Å away.

**Table 1:** Data collection and refinement statistics.

	Holo-MmNcsA
Data collection	
Wavelength (Å)	0.9793
Space group	*P*2_1_2_1_2_1_
Cell dimensions	
* a*, *b*, *c* (Å)	55.0 84.5 145.4
* α*, *β,* *γ* (°)	90, 90, 90
Resolution (Å)^a^	48.48–2.79 (2.86–2.79)
Number of unique reflections	157,172 (1150)
*R*_merge_^a^	0.183 (6.890)
*R*_pim_^a^	0.065 (2.366)
*I/*σ(*I*)^a^	7.5 (0.3)
*CC*_1/2_^a^	0.997 (0.146)
Completeness (%)^a^	99.3 (91.5)
Redundancy^a^	9.0 (9.0)
*B Wilson* (Å^2^)	94.1
Refinement	
Resolution (Å)	31.85–2.81 (2.97–2.81)
No. reflections	13,180
* R*_free_/*R*_work_	0.287/0.213 (0.216/0.223)
No. atoms	
Protein	4733
Zn/Fe	4/8
Sufate ions	45
Water	179
* B* factors (Å^2^)	
Protein	96.1
Zn/FeS	104.6/106.9
Sulfate ions	176.9
Water	62.5
R.m.s. deviations	
Bond lengths (Å)	0.008
Bond angles (°)	1.06
Ramachandran plot (molprobity)	
Favored (%)	0.866
Outliers (%)	0.029

^a^Values in parentheses are for highest-resolution shell.

Superposition of the MmNcsA and PhTtuA catalytic cores shows that the two proteins adopt the same fold but highlights different orientations of the terminal zinc-containing domains relative to the catalytic core (Fig. S4A). This indicates flexibility of the N-and C-terminal domains relative to each other, and suggests that conformational changes of these domains could occur upon tRNA binding to clamp the anticodon loop within the active site of the enzyme. The clusters of MmNcsA and PhTtuA are bound by the three conserved cysteines in the same way (Fig. S4B). Moreover, Lys135 in PhTtuA is replaced, in MmNcsA, by Arg149 that adopts two different conformations. In PhTtuA, the amino group of Lys135 is located 3.8 Å away from one sulfur atom of the cluster and is likely involved in the binding of the β phosphate of ATP^39^. The flexibility of Arg149 observed in the MmNcsA structure, in the absence of ATP/AMP bound to the active site, could reflect a similar role of Arg149 in MmNcsA as Lys135 in PhTtuA. Despite these similarities in the nearby environment of the cluster, several amino acids that belong to the second sphere of the catalytic site are different, pointing out residues potentially involved in U34-tRNA or U54-tRNA specificity. In particular, four conserved amino acids in NcsA proteins (Glu88, Arg94, Arg241, Glu267) are charged and have longer sides chains than the corresponding residues in TtuA proteins (Leu81, Ser87, Thr228, Leu254) (Fig. S4C). This implies that the uridine-binding pocket is more charged and smaller in MmNcsA compared to that of PhTtuA.

### AlphaFold model of the human Ctu1/Ctu2 complex

The recently released AlphaFold algorithm can predict very accurately not only the fold of a protein from its amino acid sequence, but also the position of its side chains when the backbone prediction is accurate^47^. In particular, for the human proteome, AlphaFold was shown to cover 58% of residues with a confident prediction (predicted local-distance difference test (pLDDT) confidence value > 70), of which a subset (36% of all residues) have a very high pLDDT confidence value (> 90)^57^.

**Figure 5:**
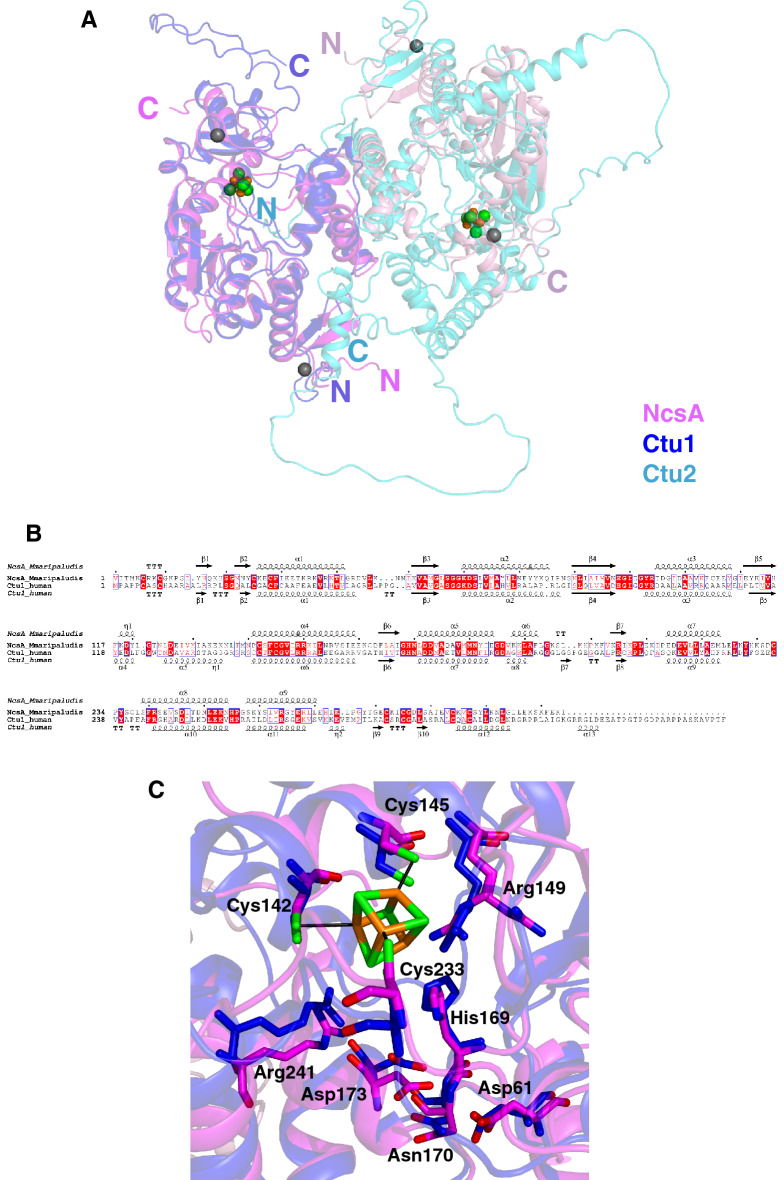
AlphaFold model of the Ctu1/Ctu2 complex. (**A**) Overview of the Ctu1/Ctu2 complex superimposed onto the MmNcsA dimer. Ctu1 from the AlphaFold model of the Ctu1/Ctu2 complex was superimposed onto one monomer of the holo-NcsA crystal structure (in magenta) with an rmsd of 1.27 Å over 229 aligned Cαs. Superposition of Ctu2 onto one NcsA monomer with the SUPER option in PYMOL gives an rmsd of 1.6 Å for 100 aligned Cαs. (**B**) Amino acid sequence alignment of MmNcsA and human Ctu1, with the secondary structures displayed above (MmNcsA crystal structure) and below (Ctu1 AlphaFold model) the alignment. (**C**) Zoom of superposition of the MmNcsA and Ctu1 active sites.

Ctu1 was shown to be physically associated with Ctu2 in fission yeast^31^, and it was suggested that both proteins form a heterodimeric complex. To get insight into the structural similarities between MmNcsA and its human Ctu1 orthologue (28.3% sequence identity), the potential human Ctu1/Ctu2 heterodimeric complex was modeled with AlphaFold (pLDDT of 80.6). Interestingly, when the Ctu1 model was superimposed onto one MmNcsA monomer of the crystal structure, then the Ctu2 model was superposed on the second NcsA monomer (Fig. 5A), indicating that the dimerization mode of the Ctu1/Ctu2 heterodimer is similar to that of the NcsA homodimer. Remarkably, the pLDDT values for the core of the Ctu1/Ctu2 heterodimer are very high (Fig. S5), indicating a highly reliable prediction. The superposition of NcsA and Ctu1 gives an excellent fit, with a Z-score of 29.3, and a root mean square deviation (rmsd) of 2.5 Å over 283 aligned Cαs out of 348, as calculated with DALI^58^. Remarkably, the same secondary structure elements are shared by MmNcsA and Ctu1, with the same orientations, the differences being located in the loops connecting these elements, and at the N and C-termini (Fig. 5B). In particular, Ctu1 exhibits a long, mainly non-structured C-terminal extremity (residues 311–348), which has no counterpart in the archaeal enzyme. Interestingly, the loop connecting residues 130 and 139 in Ctu1 is partly folded into one helix (α5) and one turn (η1), whereas it was not observed in the electron density of the MmNcsA crystal. As anticipated, in the Ctu1 model, the three conserved cysteines, Cys144, Cys147 and Cys237, are located near each other at the catalytic site, and in a similar position as the three cysteines of MmNcsA that bind the [4Fe-4S] cluster (Fig. 5C), strongly suggesting that these cysteines in Ctu1 also serve to bind a [4Fe-4S] cluster with a binding mode very likely similar to that observed in the MmNcsA crystal structure. In particular, a free coordination site at the fourth Fe is also likely, since there is no other cysteine (or other coordinating amino acid) that could provide a fourth ligand to the cluster in the Ctu1 model. Moreover, all the residues belonging to the second sphere around the cluster of MmNcsA are conserved in Ctu1 and the superposition of the equivalent residues in MmNcsA and Ctu1 highlights a high structural similarity between the two enzymes (Fig. 5C).

On the other hand, the superposition of the Ctu2 model and the NcsA crystal structure reveals that Ctu2 likely shares most secondary structure elements with NcsA/Ctu1 but also displays two additional regions, one linking α4 and α5, the other linking α12 and α13, which are poorly ordered, as shown by their pLDDT values (Fig. S6). Whereas Ctu2 does not possess the conserved cysteines that bind the [4Fe-4S] cluster in MmNcsA, excluding the presence of such a cluster in Ctu2, it still possesses the Zn finger binding sites (Fig. S6A).

## Discussion

### Various non-redox enzymes involved in tRNA thiolation

Sulfur, an essential element for a variety of cellular constituents in all living organisms, is present in several nucleosides in tRNA, such as derivatives of thiouridine (s^4^U8, s^2^U34, m^5^s^2^U54), 2-thioadenine (ms^2^i^6^A37) and 2-thiocytidine (s^2^C32)^59,60^. Two different chemical mechanisms have been proposed for tRNA thiolation enzymes catalyzing the non-redox substitution of an oxygen atom by a sulfur atom within nucleosides^61^. In all cases, the enzymes display a PP-loop motif^34,62^ characteristic of ATP-binding proteins in their sequence (Fig. S1), and activation of the target nucleoside by ATP, through the formation of a catalytically competent adenylated nucleoside, is required^35,63^.

**Figure 6:**
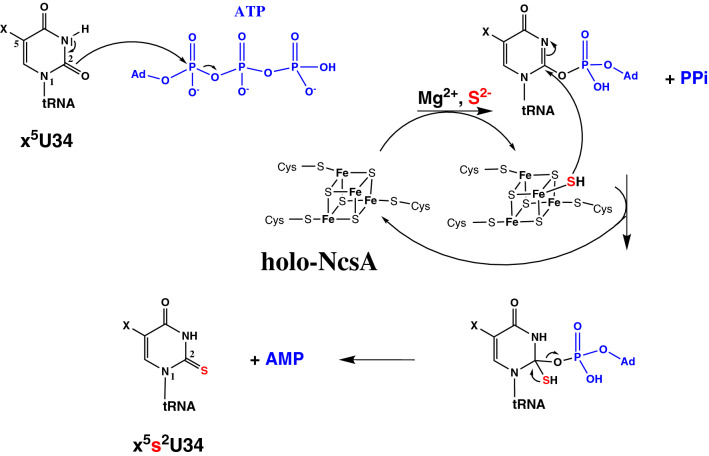
Postulated mechanism of NcsA-catalyzed U34-thiolation based on the mechanism proposed for TtuA^39^. ‘Ad’ stands for adenylate.

The first mechanism involves intermediate reactive persulfides carried by protein cysteines, as exemplified by the biosynthesis of s^4^U8 in tRNAs in *E. coli*, in which sulfur is released from free cysteine by cysteine desulfurase IscS, transferred to the rhodanese-like module of the U8-tRNA thiolase as a persulfide, which finally gives its sulfur atom, directly or indirectly, to the adenylated nucleoside^64,65^. In contrast, the biosynthesis of s^4^U8-tRNAs in several archaea^45,66^, s^2^U34-tRNA catalyzed by bacterial MnmAs^36,37^, s^2^C32-tRNA catalyzed by TtcAs^38^ and m^5^s^2^U54-tRNA catalyzed by TtuAs^39–42^ occurs via a mechanism that involves a [4Fe-4S] cluster as a cofactor. The [4Fe-4S] cluster is coordinated by three conserved amino acids only, which leaves a free coordination site on one iron atom to bind and activate the sulfur atom, coming from inorganic sulfide^39^ or a sulfur donor^41^, to promote the sulfur transfer to the adenylated substrate (Fig. 6).

### MmNcsA uses a [4Fe-4S] cluster for U34-tRNA thiolation

MnmA proteins catalyze s^2^U34 thiolation in mitochondria^30^ and bacteria, as exemplified by the well-studied *E. coli* enzyme^29,35,67^. The NcsA/Ncs6/Ctu1 family represents a class of enzymes phylogenetically distant from MnmA proteins^34^ that catalyze the same reaction in archaea and the eukaryotic cytosol (Fig. 1B). Since the two enzyme classes share a common ATPase domain, the structures of their catalytic domains can be superposed with a Z-score of 14.3 and an rmsd of 3.1 Å for 153 out of 297 Cα atoms, as calculated with DALI^58^. Otherwise, these two classes display poor sequence similarity (for example EcMnmA shares only 16.2% sequence identity with MmNcsA), and display different oligomerization modes, with *E. coli* MnmA being a monomer^35,36^ and MmNcsA a dimer*.*

Enzymes within the second class of U34-tRNA thiolases share 28–35% sequence identity (Fig. S1). Among this class, MmNcsA and human Ctu1 were previously reported to contain a [3Fe-4S] cluster when purified under anoxic conditions, with a content of Fe of 2.7 and 1 per protomer, respectively, but the catalytic activity of these enzymes was not determined^45^. The chemical nature of the cluster was intriguing because [3Fe-4S] clusters are not known to catalyze non-redox reactions^68^ and they usually come from the degradation of [4Fe-4S] clusters via loss of the fourth Fe atom^69^, especially those chelated by only three protein ligands. It was shown in several cases that the [3Fe-4S]–containing protein was inactive, indicating that the fourth Fe atom is key for catalytic activity^70,71^. To address this issue, we wished to characterize catalytically and structurally an enzyme belonging to the NcsA/Ncs6/Ctu1 class. Using UV–visible and EPR spectroscopies combined with X-ray crystallography, we show here that MmNcsA assembles a well-defined [4Fe-4S] cluster. The existence of two states of a [4Fe-4S]^+^ cluster, with different g-values, was deduced from the EPR spectrum of reduced holo-NcsA. Multiple S1/2 ground states, reflecting subtle structural differences, have previously been reported for several other [4Fe-4S] enzymes, such as *Mycobacterium tuberculosis* adenosine 5′-phosphosulfate reductase^72^, the corrinoid protein from *Clostridium thermoaceticum*^73^, human DNA primase^74^, the anaerobic ribonucleotide reductase activase from *Lactococcus lactis*^75^ and the ribosomal RNA methyltransferase RumA from *E. coli*^76^. It is known that EPR spectra of [4Fe-4S] clusters are very sensitive to small variations in their environment, resulting into different delocalization of electrons within the cluster, and thus electronic properties^50,52,53^. Thus, it is likely that the two observed forms of [4Fe-4S] cluster differ in their coordination sphere. As there is a free site on the fourth iron atom of the [4Fe-4S] cluster, one cluster form could have an empty site, whereas the second one could have a water molecule or a hydrogenosulfide ion as the fourth ligand. The latter explanation is in agreement with the S content of holo-MmNcsA (4.8 S atoms per monomer) and with the propensity of the cluster, bound to three cysteines only, to bind an exogenous molecule, as shown by the crystal structure.

We also report an in vitro anaerobic catalytic assay, which shows that MmNcsA, in its fully reconstituted [4Fe-4S] holo form, is active for tRNA thiolation. The cluster was absolutely required since apo-MmNcsA was inactive, and the reaction also required Mg^2+^_._ The control reaction lacking ATP showed some catalytic activity, suggesting the presence of some ATP-bound form of the enzyme (Fig. 3B). The control reaction lacking inorganic sulfide as a source of sulfur atoms also showed some residual catalytic activity (Fig. 3B), likely due to the presence of a small excess of sulfur atoms, originating from the chemical reconstitution of the cluster to form holo-MmNcsA, which can be mobilized in activity assays. Holo-MmNcsA, when present at a 1:10 enzyme:tRNA ratio, was able to catalyze the formation of s^2^U34 in a tRNA^Lys^_UUU_ transcript with a rate of s^2^U formation of 0.14 nmol h^−1^. The specific activity (0.023 nmol product formed min^−1^ per nmol of enzyme) is in the same range as that calculated for other [4Fe-4S]-dependent tRNA thiolation enzymes tested in similar conditions (for example 0.035 nmol min^−1^ nmol^−1^ for *E. coli* TtcA^38^, using *E. coli* Δ*ttcA* bulk tRNA as a substrate). The rather low catalytic activity of MmNcsA could be explained by several factors. First, since the formation of s^2^U34 was monitored using a tRNA^Lys^_UUU_ transcript as a substrate, higher specific activity is expected with a native tRNA substrate containing prior chemical modifications rather than a tRNA transcript. In particular, modifications at the C5 position of U34 may be needed for efficient thiolation. Indeed*,* it was shown that the mcm modification at C5 of U34 facilitated s^2^U formation in yeast tRNAs^44^. Further investigation is needed to understand the effect of prior modifications on the U34 thiolation activity. Second, in our in vitro assay, sulfur is provided in the form of a sulfide salt. Whether this is physiologically relevant or not remains a matter of discussion. A protein sulfur-relay system is used to transport sulfur from free cysteine to the U34-tRNA thiolase in several organisms^67,77,78^. In particular, in eukaryotes and some archaea, cysteine desulfurase together with a ubiquitin-like protein and activating enzymes are used to bring the sulfur from cysteine to the s^2^U34 thiolation enzyme^44,46^. Thiolation of U54-tRNA thiolase by *T. thermophilus* TtuA also involves such a sulfur-flow system composed of an ubiquitin-like TtuB protein, a TtuC protein carrying ATPase activity and a rhodanese-like TtuD protein^41,77,79^. In this case, thiocarboxylated TtuB (TtuB-COSH) is the key immediate sulfur donor for the final sulfurtransferase TtuA. Intriguingly, methanogenic archaea were reported to lack cysteine desulfurases^80^. We confirmed this analysis by a blastp search for proteins with sequence homology with cysteine desulfurases, which, indeed, could not identify a candidate in *M. maripaludis* and other *Methanococcales.* It has previously been proposed that, in these organisms, inorganic sulfide from the medium could be used as the sulfur source for the biosynthesis of [Fe-S] clusters^80^, methionine^80^, thiamin thiazole^81^ and s^4^U8-tRNA^82^, so this could also be the case for U34-tRNA thiolation. This complex issue deserves further investigation out of the scope of this study.

The discrepancy concerning the chemical nature of the cluster reported in the paper of Liu et al.^45^ and in our study may be explained by the fact that [4Fe-4S] clusters are highly air-sensitive and easily degraded to [3Fe-4S] clusters in the presence of oxygen, even at low amounts^69^. Furthermore, a [4Fe-4S] cluster, bound to three amino acids only, is expected to be even more unstable than classical [4Fe-4S] clusters with four ligands and may more easily lose one Fe atom. Therefore, it is most likely that the [3Fe-4S] cluster observed by Liu et al.^45^ represents a catalytically inactive form, as previously reported for aconitase^83^ or for the fumarate and nitrate reduction transcriptional regulator FNR^69^.

Furthermore, other [4Fe-4S]-dependent non redox enzymes have been reported^68^, such as serine dehydratase^84^ and fumarate dehydratase^71^ that catalyze reversible dehydration reactions, and thiouracil desulfidase^85^ that catalyzes a desulfuration reaction. In those cases, the cluster is bound by three cysteines only, leaving one exchangeable Fe coordination site for substrate binding. Here we show that MmNcsA not only carries a similar cluster, with three cysteine ligands and one free coordination site, but also that an exogenous ligand can bind to the latter, as revealed by the extra electron density adjacent to the unique Fe atom in the crystal structure. This was also the case for the previously solved TtuA structure for which we attributed the electron density to a hydrogenosulfide ion^39^. Hydrogenosulfide bound to a [4Fe-4S]^2+^ cluster has also previously been characterized in the crystal structure of 2-hydroxyisocaproyl-CoA dehydratase^86^ and thiouracil desulfidase^85^. The propensity of the fourth non protein-bonded iron to bind a small molecule indicates that the [4Fe-4S] cluster likely plays a direct role as a Lewis acid in catalysis, specifically to bind, activate a HS^−^ species and transfer it to the activated substrate, as proposed earlier for TtuA^39^ (Fig. 6).

### U34-tRNA biosynthesis is most likely performed by a heterodimeric complex in eukaryotes

Deciphering the mechanism of eukaryotic U34-tRNA thiolases is of outmost importance as the Ctu1/Ctu2 complex is required to maintain genome integrity. Indeed, disruption of the Ctu1/Ctu2 complex caused abnormal phenotypes in worms, yeast and plants^31,78,87^. It has also been shown that U34 modifying enzymes are up-regulated in human breast cancers^88^ and that they promote the survival and resistance to therapy of melanoma cells by regulating specific mRNA translation^28^. Hence, targeting U_34_ modifying enzymes could represent a new therapy to treat melanoma patients^89^.

Given the high sequence identity between archaeal and eukaryotic NcsA/Ncs6/Ctu1 proteins, including the conservation of the cysteines that coordinate the [4Fe-4S] in MmNcsA (Fig. S1), together with their structural similarity (Fig. 5), we propose that eukaryotic proteins also bind a [4Fe-4S] cluster and share the same mechanism as archaeal NcsA enzymes (Fig. 6).

Since the human Ctu1/Ctu2 complex has not been purified and characterized yet, the stoichiometry of the proteins composing such a complex has not been confirmed experimentally. However, both proteins are required for tRNA thiolation in vivo^31,44,78,87^, whereas the archaeal enzyme is active on its own. Interestingly, the AlphaFold model of the Ctu1/Ctu2 heterodimer is very close to the crystal structure of the MmNcsA homodimer, strongly suggesting that a Ctu1/Ctu2 heterodimer may exist by itself or is part of a complex of higher oligomeric order. Moreover, the models of Ctu1 and Ctu2 are very close to each other, suggesting that the two proteins share a common origin (Fig. 5). Hence, the human U34-tRNA thiolase appears to be the product of gene duplication and divergent evolution of a homodimeric archaeal enzyme, with the catalytic subunit Ctu1 containing the conserved active site, whereas the second subunit Ctu2 diverged, most likely to ensure the correct orientation of the tRNA substrate. Analogous evolutionary scenarios have been suggested for eukaryotic and archaeal/prokaryotic tRNA modifying enzymes and tRNA splicing enzymes. Indeed, eukaryotic A58-tRNA methyltransferases are heterotetramers composed of two distantly diverged but structurally homologous subunits^90^. Since the tRNA molecule is asymmetric, it was proposed that the use of different subunits in the heteromultimeric enzyme allows more flexible optimization of the binding surface and provides efficient means to circumvent the problem of concerted amino acid substitutions that occur in structurally identical, but functionally non-equivalent subunits of the homomultimeric proteins. Another example is the heterodimeric eukaryotic ADAT2/ADAT3 adenosine-34-tRNA deaminase that has a single counterpart in *E. coli*^91^. Similarly, it is believed that the heterotetrameric yeast tRNA Sen nuclease evolved from the homotetrameric archaeal EndA enzyme, in which the structurally identical subunits have non-equivalent roles in tRNA binding and catalysis^92^. Therefore, it seems that replacement of homomultimeric tRNA enzymes with heteromultimers encoded by duplicated and diverged genes is a common evolutionary mechanism in eukaryotic organisms^93^.

## Material and methods

### Cloning of the *ncsA* gene

The gene encoding the NcsA protein from *M. maripaludis* (Mmp1356) was synthesized by Eurofins with codon optimization for expression in *E. coli* and sub-cloned into the pBG102 plasmid (pET27 derivative) between the BamHI and EcoRI restriction sites to produce a 6His-SUMO-NcsA protein construct whose 6His-SUMO tag can be cleaved by the human rhinovirus 3C protease.

### Overexpression of MmNcsA

The plasmid containing the *ncsA* gene was transformed in *E. coli* BL21(*DE3*) competent cells. One colony was used to inoculate 200 mL of Luria Broth (LB) medium supplemented with kanamycin (50 mg/L). 120 mL of this preculture, grown overnight at 37 °C, was used to inoculate 6 L of LB medium supplemented with the same antibiotic. Cultures were grown at 37 °C to an OD_600_ of 1–1.2, and expression was induced by addition of isopropyl-β-d-thiogalactopyranoside (IPTG) to a final concentration of 0.5 mM for 3 h. After overnight incubation at 25 °C, cells were collected by centrifugation and stored at − 20 °C.

### Purification of MmNcsA

Cells were resuspended in 60 mL of 50 mM NaH_2_PO_4_ pH 7, 500 mM NaCl, 40 mM imidazole containing RNase A (2 µg/mL) and benzonase (100 U, Sigma Aldrich), and disrupted by sonication. Cells debris were removed by centrifugation at 210,000 *g* for one hour at 4 °C. The supernatant was then loaded on an immobilized metal affinity Ni–NTA column (HisTrap 5 mL, Cytiva) equilibrated in 50 mM NaH_2_PO_4_ pH 7, 500 mM NaCl, 40 mM imidazole and eluted with a linear gradient of 0.04–0.5 M imidazole. The protein was collected, dialyzed overnight against 5 L of 50 mM Tris–HCl pH 7.5, 150 mM NaCl in the presence of the 3C Protease (25 µg/mg NcsA), centrifugated at 4 °C for 10 min then loaded at 1 mL min^−1^ onto a MonoS 5/50 cation exchange column (Cytiva) using an ÅKTA system. Elution was performed with a linear gradient of 0.02–0.5 M NaCl in 50 mM Tris–HCl pH 7.5 for 20 min. Fractions containing the NcsA protein were concentrated and loaded onto a gel filtration column (Hiload 16/60 Superdex 75, Cytiva), in 25 mM HEPES pH 7.5, 200 mM NaCl, 5 mM dithiothreitol (DTT). The as-purified protein was concentrated to 10 mg/mL with an Amicon Ultra filter device (30 kDa cutoff, Millipore), frozen in liquid nitrogen and stored at − 80 °C.

The GST–3C-protease (PreScission, a gift from S. Mouilleron) was expressed using pGEX-2T recombinant plasmids. After induction at 25 °C with 0.1 mM IPTG for 20 h, the protein was purified using glutathione–Sepharose chromatography.

### [Fe-S] cluster reconstitution and purification of holo-MmNcsA

To remove the residual cluster and form apo-MmNcsA, 5 mg of as-purified MmNcsA was incubated in the presence of 2 mM dithionite and 10 mM EDTA for 2 h at 17 °C under strict anaerobic conditions in an Mbraun glove box containing less than 0.5 ppm O_2_, then desalted on a PD10 column equilibrated in 25 mM HEPES pH 7.5, 200 mM NaCl. The reconstitution of the [4Fe-4S] cluster and purification of holo-MmNcsA were then performed in the glove box. After incubation of 100 µM apo-MmNcsA with 5 mM dithiothreitol for 15 min, a fivefold molar excess of ferrous ammonium sulfate and L-cysteine as well as 2 µM *E. coli* cysteine desulfurase CsdA were added, and incubation was extended overnight. After centrifugation for 20 min at 20,000*g*, holo-MmNcsA was loaded onto a Superdex 200 10/300 gel filtration column (Cytiva) equilibrated in 25 mM HEPES pH 7.5, 200 mM NaCl, 5 mM DTT. The peak containing the holo-MmNcsA dimer was then concentrated to 15–25 mg/mL on a Vivaspin concentrator (30 kDa cutoff).

### Quantification methods

The Pierce BCA assay was used to quantify the protein^94^. The Fish and Beinert methods were routinely used after cluster reconstitution to quantify iron and sulfide, respectively^48,49^.

### Preparation of bulk tRNA and in vitro transcribed Mm-tRNA^Lys^_UUU_

Bulk tRNA from the GRB 105 Δ*ttcA E. coli* strain was purified as reported^95^. Mm-tRNA^Lys^_UUU_ was synthesized in vitro by T7-RNA polymerase transcription as described^96^. Before use, the tRNA transcript was refolded by heating at 65 °C for 15 min then cooling slowly to 45 °C.

### In vitro enzyme assay

1 µM holo-MmNcsA (or 10 µM holo-MmNcsA) and 20 µM Mm-tRNA^Lys^_UUU_ were incubated at 37 °C in 100 µl of 25 mM HEPES pH 7.5, 200 mM NaCl in the presence of 0.25 mM ATP, 2.5 mM MgCl_2_ and 0.25 mM Na_2_S under anaerobic conditions for 0 to 120 min. The reaction was stopped by adding 2.5 µL 3 M formic acid, and loading dye. The thiolated and unmodified tRNAs were separated on a 12% urea Poly-Acrylamide-Gel-Electrophoresis gels supplemented with 15 μg/ml APM^54^ then visualized by staining with 0.1% [w/v] toluidine in 40% [v/v] methanol and 1% [v/v] acetic acid.

### tRNA digestion and analysis of modified nucleosides

20 µM tRNA was digested overnight in 100 µL of 25 mM HEPES pH 7.5, 200 mM NaCl, 0.1 mM ZnSO_4_ at 37 °C by nuclease P1 (2 units, Sigma) followed by the addition of alkaline phosphatase for 2 h at 37 °C (2 units, Sigma). HPLC-tandem mass spectrometry analyses were performed with an Accela chromatographic system coupled with a Quantum ultra-triple quadripolar apparatus (Thermo Electron SAS) equipped with an HESI electrospray source used in the positive ionization mode. HPLC separation was carried out with a 2 × 150 mm octadecylsilyl silica gel (3-mm particle size) column (Uptisphere, Interchim Montluçon, France) and a 0 to 15% linear gradient of acetonitrile in 2 mM ammonium formate over 20 min as the mobile phase. Mass spectrometry detection was carried out in the multiple reactions monitoring mode to obtain high sensitivity and specificity. The transitions used to quantify the nucleosides were 261 → 129 for s^2^U and 303 → 171 for mnm^5^s^2^U, corresponding to the loss of ribose. Quantification was performed by external calibration.

### Characterization of the [4Fe-4S] cluster by UV–visible spectroscopy and EPR

UV–visible absorption spectra were recorded in quartz cuvettes (1 cm optic path) under anaerobic conditions in a glove box on a XL-100 Uvikon spectrophotometer equipped with optical fibers. EPR spectra of 400 µM holo-MmNcsA, untreated or treated with 10 mM sodium borohydride in 25 mM Tris–HCl pH 8, 200 mM NaCl, were recorded on a Bruker ELEXSYS-E500 continuous-wave EPR spectrometer operating at 9.39 GHZ, equipped with an SHQE cavity cooled by a helium flow cryostat (ESR 900 Oxford Instruments). The EPR spectra of the frozen solutions were recorded at 20 K under non-saturating conditions using a microwave power of 2 mW, a modulation amplitude of 6 G, a modulation frequency of 100 kHz, and an accumulation of 4 scans. The simulation of the EPR spectrum was performed with the Easyspin software (http://www.easyspin.org/).

### Crystallization, data collection and structure determination

Holo-MmNcsA at 10 mg/mL was crystallized under anaerobic conditions by the hanging drop procedure by mixing 1 µL protein with 1 µL reservoir solution containing 0.1 M HEPES pH 7.5, 0.2 M ammonium sulfate, 18% (w/v) PEG 4000, 10% isopropanol at 19 °C. X-ray data were collected on a single crystal at 100 K at the SOLEIL synchrotron (Saint Aubin, France) on the PROXIMA-2 beamline. Data were indexed, processed and scaled with XDS^97^. Noticeable anisotropy in the diffraction was taken into account, and corrected by the STARANISO program, accessible by the server http://staraniso.globalphasing.org. The *P. horikoshii* apo-TtuA protein (PDB code 3VRH), in which the C-terminal end was truncated and amino acids different from those of NcsA were changed to alanine, was used as a model to solve the structure by molecular replacement with *PHASER*^98^. *BUSTER*^99^ was used for refinement and *COOT*^100^ for model reconstruction. Omit maps were calculated by omitting the ligand and using the *MapOnly* option in *BUSTER*. Data collection and refinement statistics are given in Table 1. In both molecules of the asymmetric unit, two loops (residues 130–137 and 275–280), for which there was no electron density, were not modelled.

### AlphaFold modeling

The AlphaFold models of the Ctu1/Ctu2 complex were calculated with the Google Colab platform and AlphaFold2_advanced option https://colab.research.google.com/github/sokrypton/ColabFold/blob/main/beta/AlphaFold2_advanced.ipynb#scrollTo=ITcPnLkLuDDE that does not use templates (homologous structures), and refined using the Amber-relax option to enhance the accuracy of the side chains geometry. The default mode of sampling options was used: num_models = 5, ptm option, num_ensemble = 1, max_cycles = 3, tol = 0, num_samples = 1, assembly option 1:1 value. The models were ranked according to their pLDDT values (higher = better).

## Supplementary Information

Supplementary Figures.

## Data Availability

Coordinates and structure factors have been deposited in the Protein Data Bank under accession code 6SCY. The Ctu1/Ctu2 model calculated with AlphaFold is accessible via the following link 10.13140/RG.2.2.11145.72807.
