# Supplementary material for: The thiolation of uridine 34 in tRNA, which controls protein translation, depends on a [4Fe-4S] cluster in the archaeum Methanococcus maripaludis
Source: Sci Rep. 2023 Apr 1;13:5351. doi: 10.1038/s41598-023-32423-9 (PMC10067955; doi:10.1038/s41598-023-32423-9)
Supplement: Supplementary file 1 — Supplementary Figures. [file 41598_2023_32423_MOESM1_ESM.pdf]

Supplementary data file

The thiolation of uridine 34 in tRNA, which controls protein translation, depends on a [4Fe-4S] cluster in the archaeum *Methanococcus maripaludis*.

Ornella Bimai[1], Pierre Legrand[2], Jean-Luc Ravanat[3], Nadia Touati[4], Jingjing Zhou[1], Nisha He[1], Marine Lénon[5], Frédéric Barras[5], Marc Fontecave[1], Béatrice Golinelli-Pimpaneau[1]*

Figure S1: Amino acid sequence alignment of several members of the TtuA/NcsA proteins subfamily. The sequences of several NcsA/Ncs6/Ctu1 orthologues (MmNcsA [1], *Haloferax volcanii* NcsA (HVO_0580) [2], *S. cerevisiae* Ncs6 [3], human Ctu1 [4], *Caenorhabditis elegans Ctu1* [5], *Arabidopsis thaliana* Rol5 [6] and Ncs2/Ctu2 orthologues (*S. cerevisiae* Ncs2 [3], human Ctu2 [4], *C. elegans* Ctu2 [5], *A. thaliana* Ctu2 [7] were aligned with PhTtuA [8] using Clustal Omega [9] and the alignment was visualized with *ESPript* [10]. The secondary structure elements of MmNcsA and PhTtuA are shown above and below the alignment, respectively. All enzymes contain three conserved cysteines (indicated as blue spheres) that were shown to coordinate the [4Fe-4S] cluster in TtuA and NcsA. The subfamily also contains two zinc finger motifs at the N- and C-termini with the residues coordinating the Zn^{2+} ions highlighted by magenta spheres.

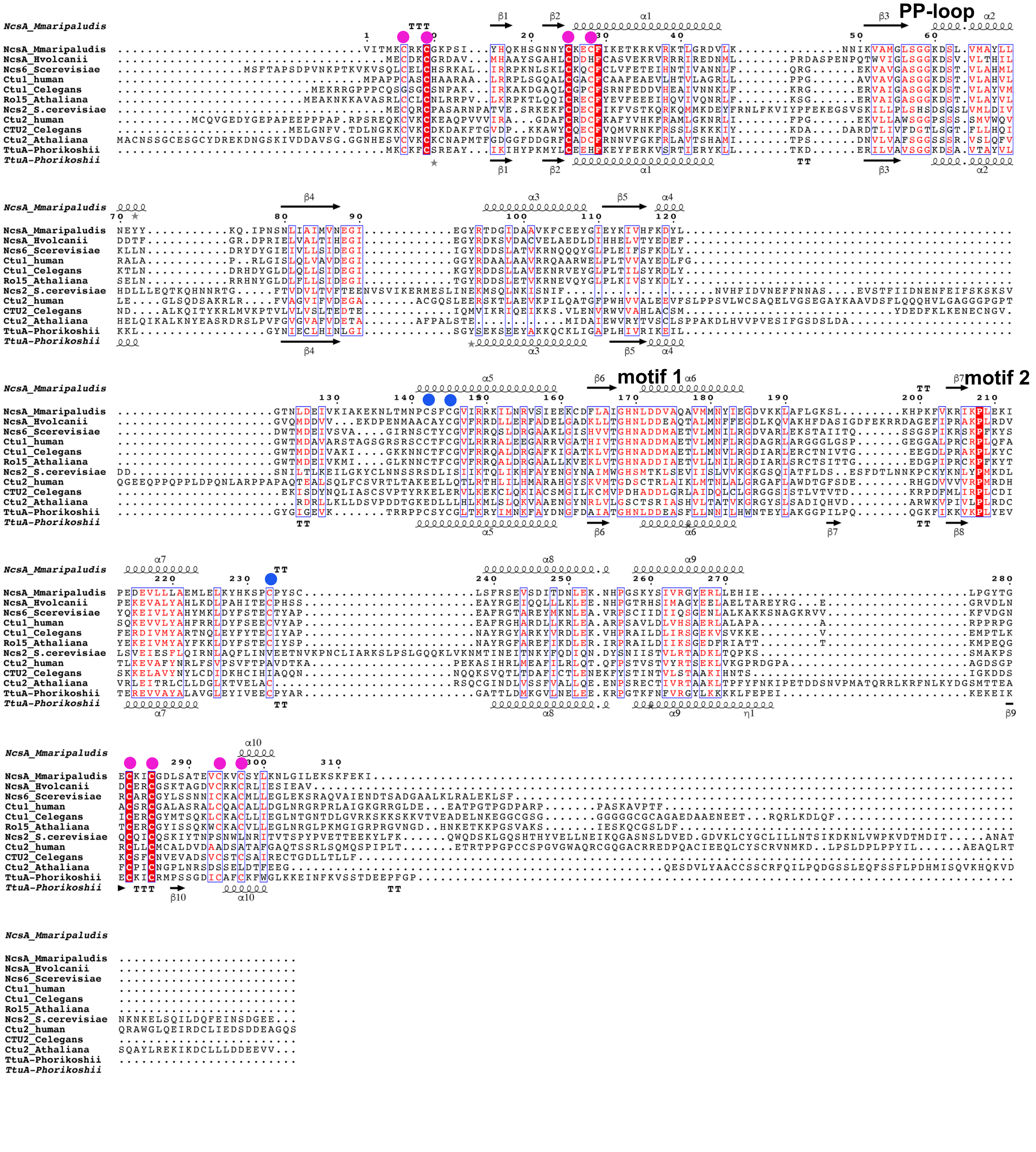

Figure S2. Purification of MmNcsA

A Purification of MmNcsA under aerobic conditions on a Superdex S200 10/300 gel filtration column. **B** 15% SDS-PAGE gel of as-purified MmNcsA. **C** UV-visible absorption spectrum of 40 μM as-purified MmNcsA. **D** Purification of apo-MmNcsA on a Superdex 75 10/300 Increase gel filtration column. Given an elution volume of 9.0 ml, a calibration curve (insert) with Biorad standards (γ-globulin, 158 kDa; Ovalbumin, 44 kDa; Myoglobin, 17 kDa; and Vitamin B12 (1,36 Da) gave a molar mass of 73.4 kDa for apo-MmNcsA, consistent with a globular dimeric state (theoretical molar mass of monomer: 36.1 kDa). **E** Purification of holo-MmNcsA under anaerobic conditions on a Superdex 200 10/300 gel filtration column. **F** 15% SDS-PAGE gel of holo-MmNcsA.

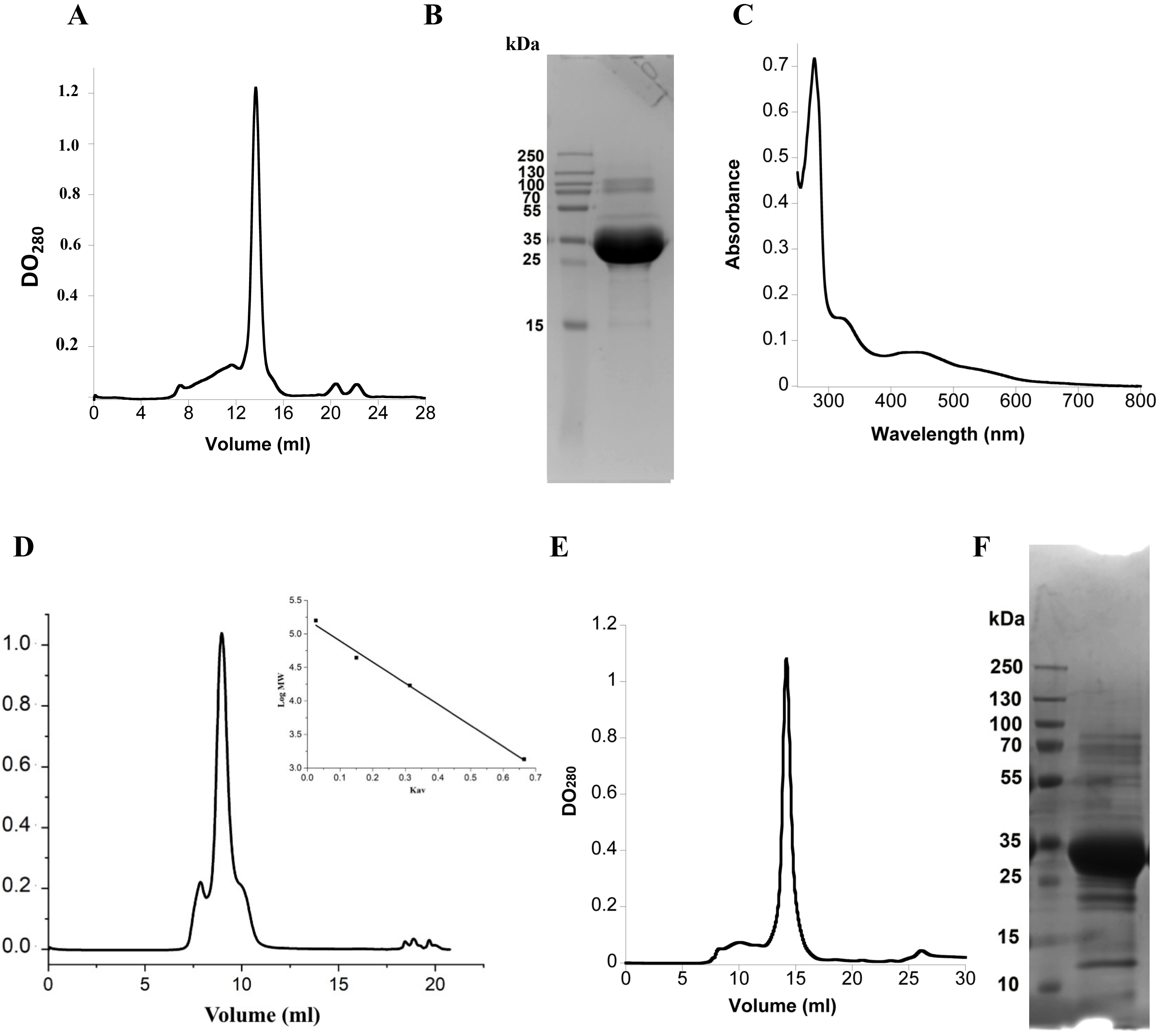

Figure S3. HPLC-MS/MS detection of the s^2U nucleoside (m/z 261). The three most intense transitions were monitored for quantitative analysis, with the 261->112 transition corresponding to the loss of sugar (in blue). Left: s^2U standard; middle: Mm-tRNA^{Lys} alone after hydrolysis, right: Mm-tRNA^{Lys} after incubation with MmNcsA in the presence of Na_2S, ATP, $MgCl_2$ and hydrolysis.

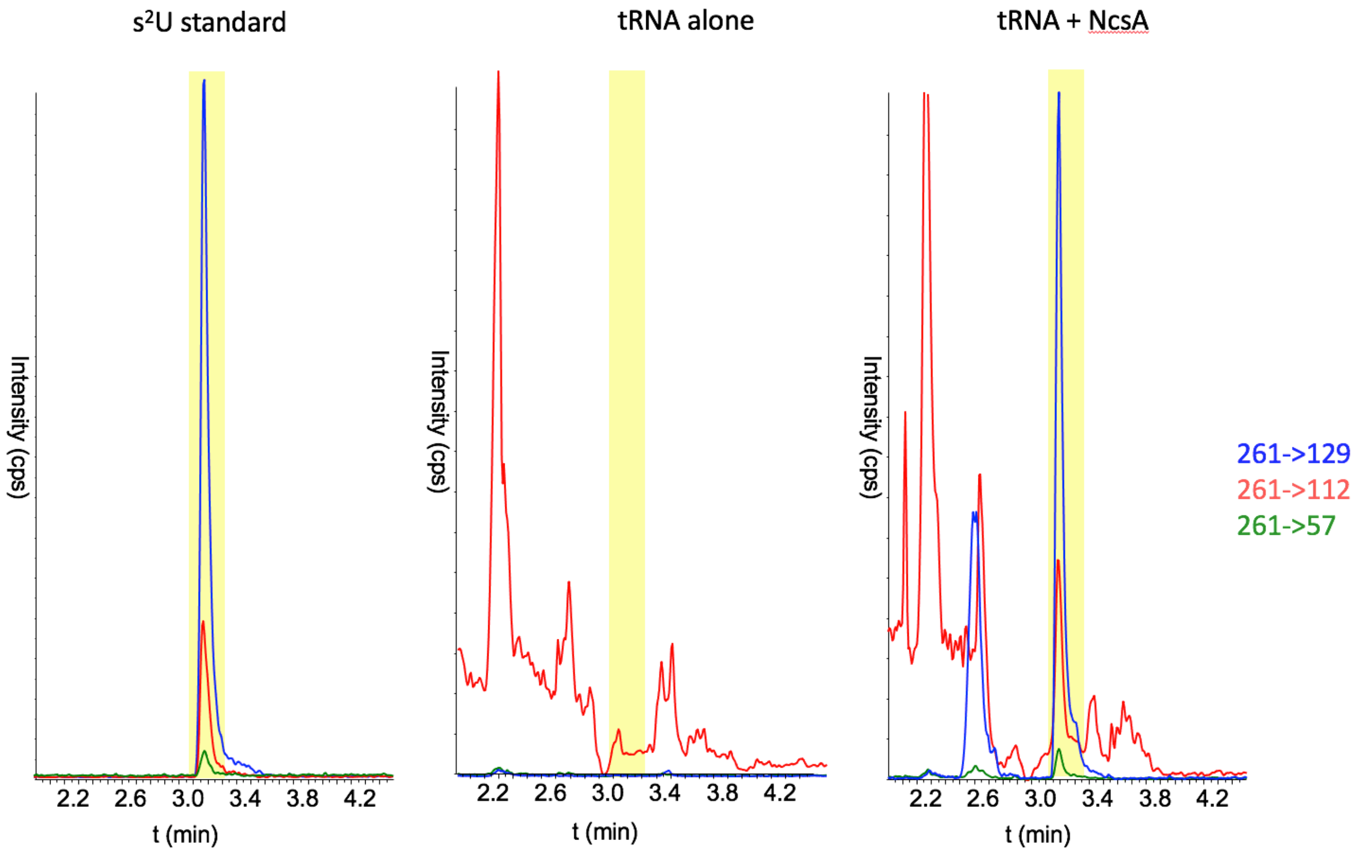

Figure S4. Superposition of the holo-PhTtuA and holo-MmNcsA structures. Residues 49-70 of PhTtuA-A and 51-72 of MmNcsA-A, corresponding to the PP-loop motif (shown as ribbon), were superimposed (rmsd of 0.544 Å for 21 Cα atoms). The cluster of MmNcsA is shown as orange (iron) and yellow spheres (sulfur). The zinc atoms are shown as spheres with color corresponding to the molecule they belong to: MmNcsA-A, pink; MmNcsA-B, magenta; PhTtuA-A, cyan; PhTtuA-B, blue. **A** Overall view of the superposition. **B** Zoom of the superposition of the [4Fe-4S] cluster bound by 3 cysteines only, at the active site of holo-PhTtuA and holo-MmNcsA. **C** Stereoview of the superposition of the active sites of holo-PhTtuA and holo-MmNcsA. Only residues from MmNcsA are labeled.

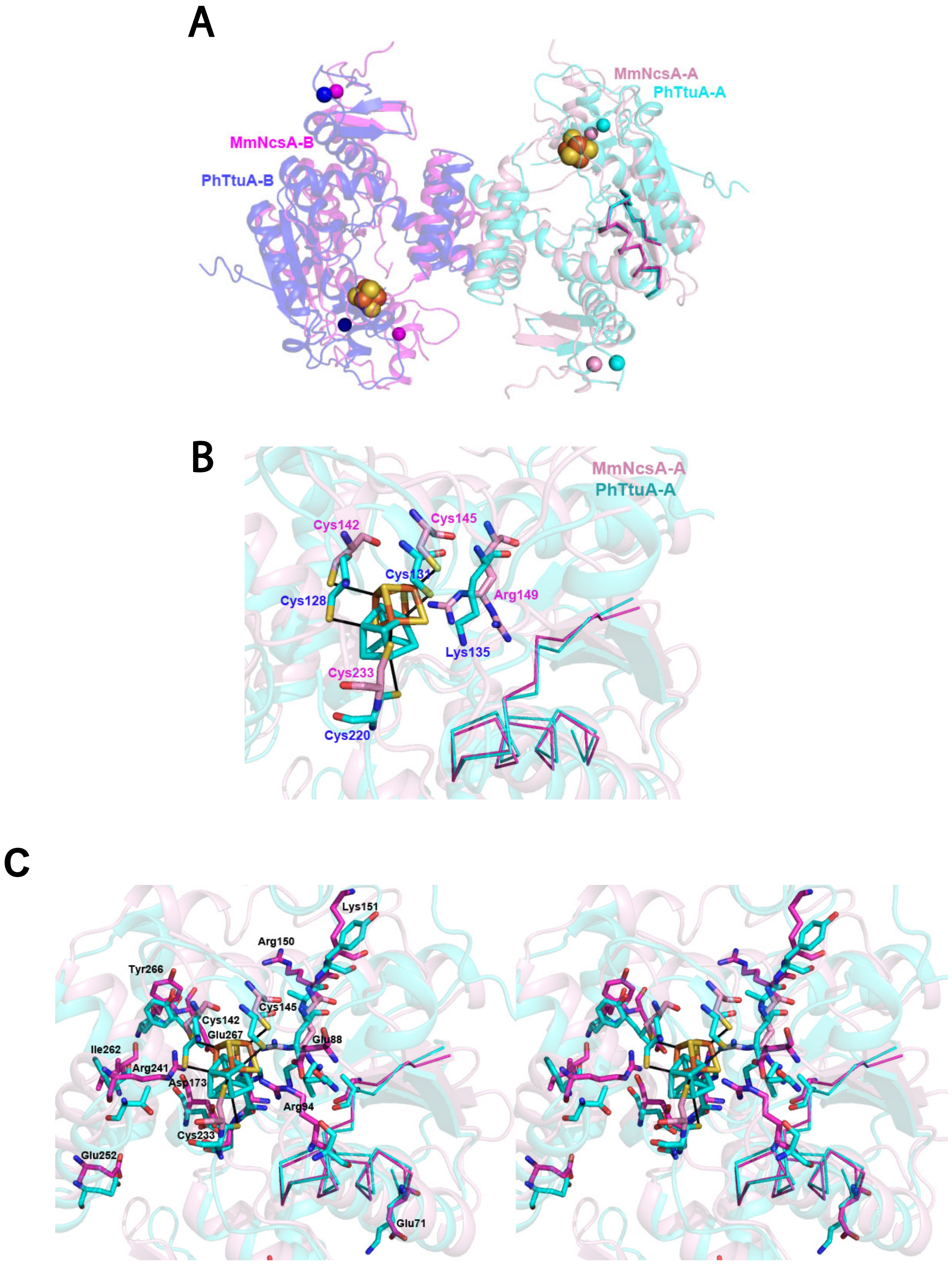

Figure S5: AlphaFold model of the Ctu1/Ctu2 complex. Both Ctu1 and Ctu2 are colored according to the pLDDT values of their backbone carbon atoms, from red (pLDDT of 20) to blue (pLDDT of 100). The orientation is the same as in Figure 5A. The N and C termini are indicated and colored in blue and cyan for Ctu1 and Ctu2, respectively.

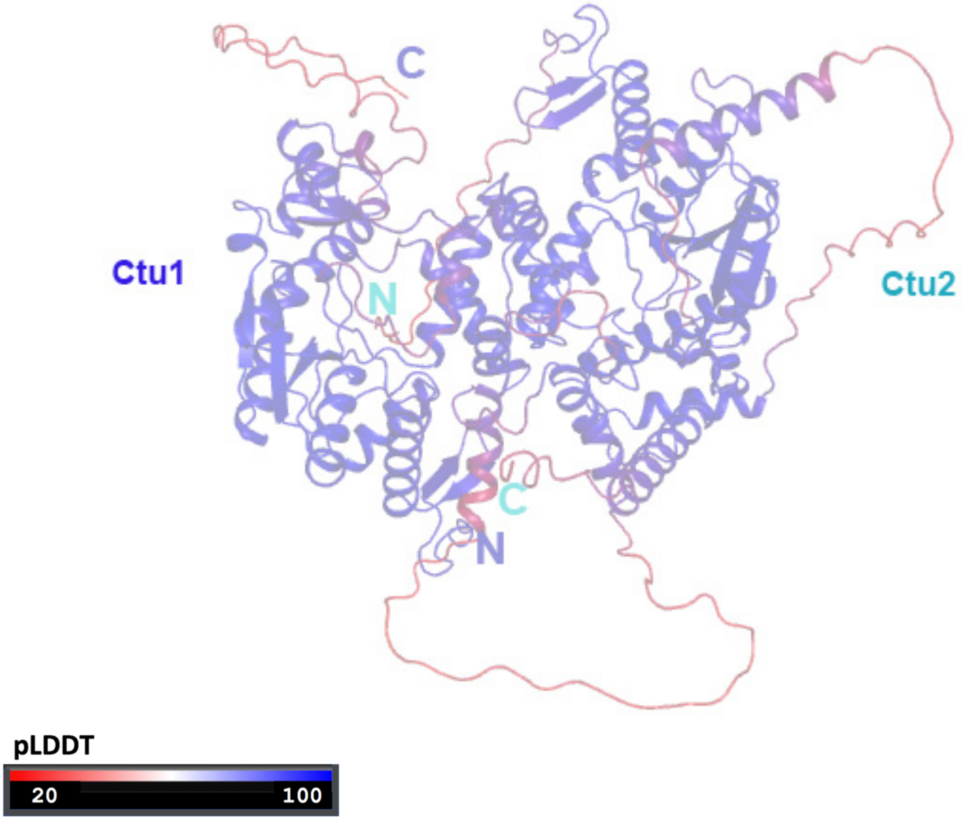

Figure S6: **A** Amino acid sequence alignment of MmNcsA and human Ctu2, with the secondary structures displayed above (MmNcsA crystal structure) and below (Ctu2 AlphaFold model) the alignment. **B** The Ctu2 AlphaFold model was superimposed with PYMOL onto one MmNcsA monomer of the crystal structure with an rmsd of 1.2 Å for 60 aligned Cα atoms. MmNcsA is shown in pink and Ctu2 is colored according to the pLDDT values of its backbone carbon atoms, from red to blue. The [4Fe-4S] cluster and Zn atoms of MmNcsA are shown as spheres.

A

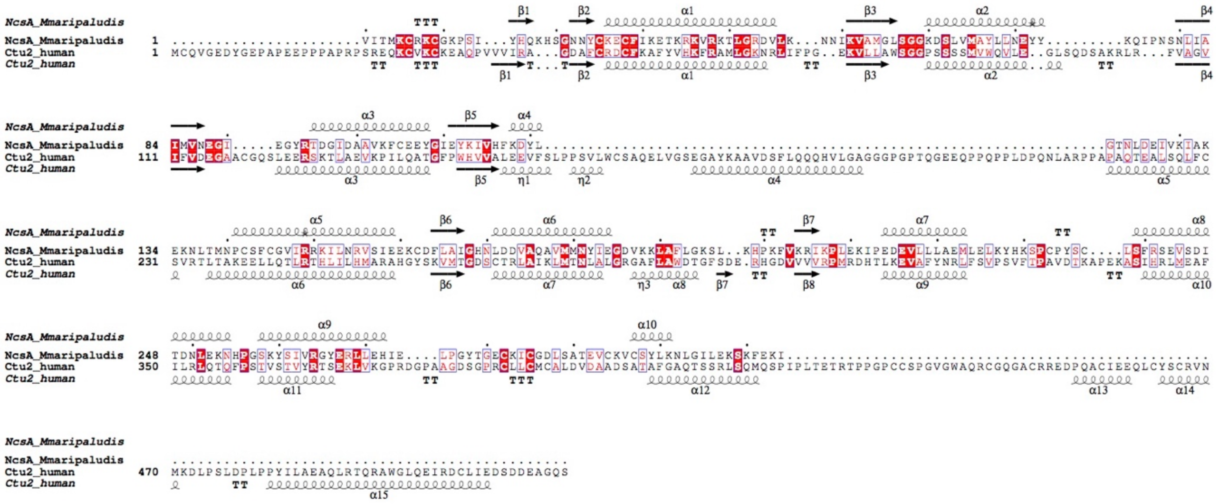

B

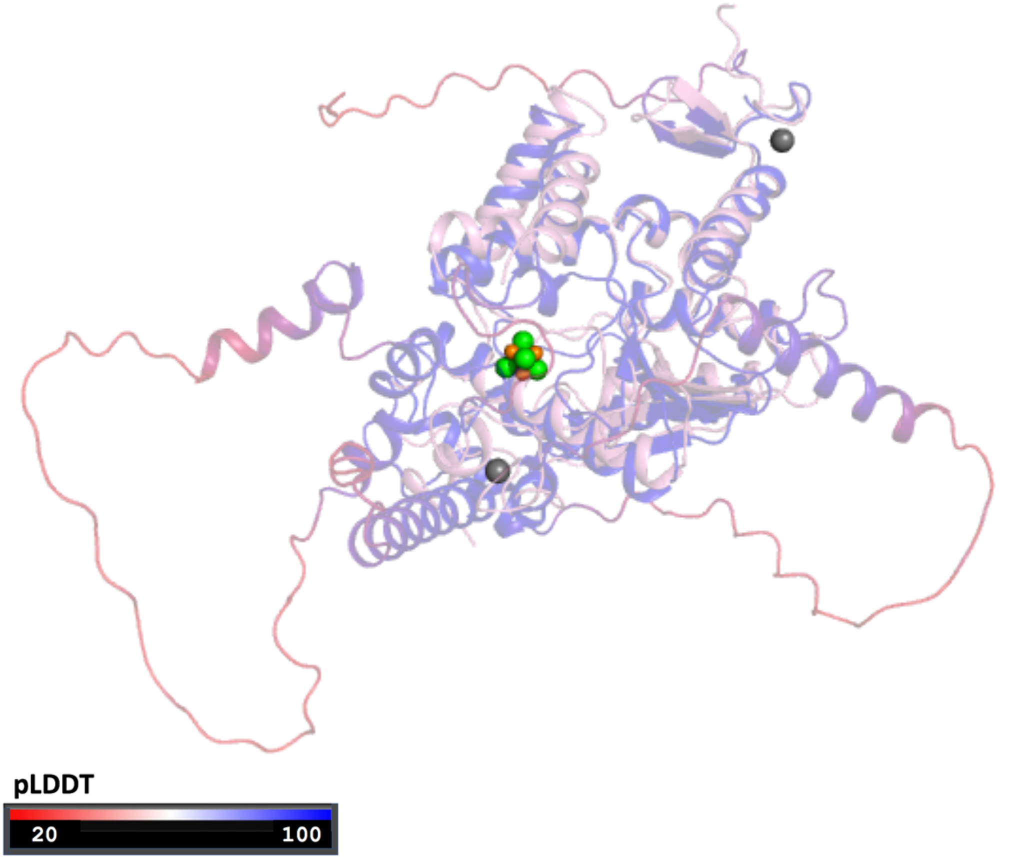

1. Liu Y, Long F, Wang L, Söll D, Whitman WB. The putative tRNA 2-thiouridine synthetase Ncs6 is an essential sulfur carrier in *Methanococcus maripaludis*. *FEBS Lett* 588, 873-877 (2014).
2. Chavarria NE*, et al.* Archaeal Tuc1/Ncs6 homolog required for wobble uridine tRNA thiolation is associated with ubiquitin-proteasome, translation, and RNA processing system homologs. *PLoS One* 9, e99104 (2014).
3. Noma A, Sakaguchi Y, Suzuki T. Mechanistic characterization of the sulfur-relay system for eukaryotic 2-thiouridine biogenesis at tRNA wobble positions. *Nucleic Acids Res* 37, 1335-1352 (2009).
4. Chowdhury MM, Dosche C, Lohmannsroben HG, Leimkuhler S. Dual role of the molybdenum cofactor biosynthesis protein MOCS3 in tRNA thiolation and molybdenum cofactor biosynthesis in humans. *J Biol Chem* 287, 17297-17307 (2012).
5. Dewez M, Bauer F, Dieu M, Raes M, Vandenhaute J, Hermand D. The conserved Wobble uridine tRNA thiolase Ctu1-Ctu2 is required to maintain genome integrity. *Proc Natl Acad Sci U S A* 105, 5459-5464 (2008).
6. Leiber RM, John F, Verhertbruggen Y, Diet A, Knox JP, Ringli C. The TOR pathway modulates the structure of cell walls in *Arabidopsis*. *Plant Cell* 22, 1898-1908 (2010).
7. Philipp M, John F, Ringli C. The cytosolic thiouridylase CTU2 of *Arabidopsis thaliana* is essential for posttranscriptional thiolation of tRNAs and influences root development. *BMC Plant Biology* 14, 109 (2014).
8. Nakagawa H*, et al.* Crystallographic and mutational studies on the tRNA thiouridine synthetase TtuA. *Proteins* 81, 1232-1244 (2013).
9. Sievers F*, et al.* Fast, scalable generation of high-quality protein multiple sequence alignments using Clustal Omega. *Mol Syst Biol* 7, 539 (2011).
10. Gouet P, Courcelle E, Stuart DI, Metoz F. ESPript: analysis of multiple sequence alignments in PostScript. *Bioinformatics* 15, 305-308 (1999).
